# Role of Histone Demethylases in Cardiomyocytes Induced to Hypertrophy

**DOI:** 10.1155/2016/2634976

**Published:** 2016-09-18

**Authors:** Wendy Rosales, Juan Carulla, Jeison García, Diana Vargas, Fernando Lizcano

**Affiliations:** ^1^Center of Biomedical Research Universidad de La Sabana (CIBUS), Chía, Colombia; ^2^Fundación Cardioinfantil, Instituto de Cardiología, Bogotá, Colombia

## Abstract

Epigenetic changes induced by histone demethylases play an important role in differentiation and pathological changes in cardiac cells. However, the role of the jumonji family of demethylases in the development of cardiac hypertrophy remains elusive. In this study, the presence of different histone demethylases in cardiac cells was evaluated after hypertrophy was induced with neurohormones. A cell line from rat cardiomyocytes was used as a biological model. The phenotypic profiles of the cells, as well as the expression of histone demethylases, were studied through immunofluorescence, transient transfection, western blot, and qRT-PCR analysis after inducing hypertrophy by angiotensin II and endothelin-1. An increase in fetal gene expression (ANP, BNP, and *β*-MHC) was observed in cardiomyocytes after treatment with angiotensin II and endothelin-1. A significant increase in JMJD2A expression, but not in UTX or JMJD2C expression, was observed. When JMJD2A was overexpressed in cardiomyocytes through transient transfection, the effect of neurohormones on fetal cardiac gene expression was increased. We conclude that JMJD2A plays a principal role in the regulation of fetal cardiac genes, which increase in expression during the pathological hypertrophic process.

## 1. Introduction

Cardiac cells undergo hypertrophic growth in response to physiological events such as pregnancy and exercise. However, when hemodynamic stress is a consequence of pathologies such as diabetes mellitus type 2, cardiac ischemia, obesity, or hypertension, cardiac cells undergo genetic and epigenetic changes that stimulate a pathology-induced stress program that includes increased expression of brain natriuretic peptide (BNP), atrial natriuretic peptide (ANP), and *β*-myosin heavy chain (*β*-MHC), which accomplish pathological and adaptive roles [1–3]. Neuroendocrine signals mediated by angiotensin II (Ang II) and endothelin-1 (ET-1) induce cardiac hypertrophy by activating many intracellular signaling cascades. An increase in intracellular Ca^2+^ activates calcium/calmodulin-dependent protein kinase (CaMKII) or calcineurin, which is the common final point that mediates cardiomyocyte growth [4]. The pathologically hypertrophic heart loses plasticity with reduced adaptive capacity, increased rates of cardiomyocyte death, fibrotic remodeling, and decreased systolic and diastolic function that often progresses towards heart failure [5]. Epigenetic modifications influenced by the environment have produced a special interest in finding the origin of cardiac hypertrophy. Within epigenetic modifications, changes in methylation of the lysine residues in the N- terminal tails of histones H3 and H4 are responsible for controlling gene expression [6–8]. Therefore, a balance of the methylation/demethylation of lysine residues in histones is important for gene expression and genomic integration [9, 10]. Both the localization of the lysine residues on histone tails and the degree of methylation (mono-, di-, or trimethylation) modify the state of differential gene expression [10–12]. Evidence suggests that the deregulation of these lysine methylation properties may play a causative role in promoting congenital heart diseases and adult cardiac hypertrophy [13, 14].

Many histone demethylases have been studied in connection with the development of hypertrophy and other cardiovascular diseases. However, the lysine histone demethylase oxygenase (JMJD/KDM) family probably has a greater impact on such diseases [15, 16]. Lysine-specific demethylase 4A (JMJD2A/KDM4A) was the first tridemethylase discovered [17], with the capacity to demethylate the H3K9m_3_ and H3K36m_3_, which can increase gene expression [17–19]. It has been shown that JMJD2A may promote cardiac hypertrophy in mouse models under pathological conditions [20]. Other lysine demethylases such as UTX/KDM6A, JMJD3/KDM6B, and JMJD2C/KDM4C have important roles in the differentiation of cardiac cells from their regulating progenitors, but their role in cardiac hypertrophy is elusive [21, 22]. In the present work, the role of neurohormones ET-1 and Ang II in inducing cardiac hypertrophy was examined and the expression of some histone demethylases was evaluated. JMJD2A was the only histone demethylase observed to significantly increase after cardiac hypertrophy induction. These studies showed that the overexpression of JMJD2A may even stimulate an increase in fetal cardiac gene expression.

## 2. Materials and Methods

### 2.1. Cell Culture

For the development of the hypertrophy model, the H9C2 cell line from the American Tissue Culture Collection (ATCC; Manassas, VA, USA) was used. The cells were grown at 37°C with 5% CO_2_ and maintained in Dulbecco's Modified Eagle Medium: Nutrient Mixture F-12 (DMEM/F-12-GlutaMAX*™*) (GIBCO, Life Technologies, Grand Island, NY, USA) supplemented with 1% Pen Strep and 10% Fetal Bovine Serum (FBS) (GIBCO, Life Technologies, Grand Island, NY).

### 2.2. Hypertrophic Induction

The hypertrophic induction of rat H9C2 cardiomyocytes took place in six-well plates when the cell culture reached 80% confluence. The concentration of FBS in the culture medium was reduced to 2% and Ang II at 200 nM (Sigma-Aldrich, Saint Louis, MO, USA) and ET-1 at 100 nM concentration were added (Sigma-Aldrich, Saint Louis, MO, USA).

### 2.3. Immunofluorescence Assays

Cardiomyocytes were grown on coverslips and treated with ET-1 and Ang II. Once hypertrophy was induced, the samples were preextracted with 0.2% PBS/Triton X-100 and fixed for 15 minutes in 3.7% formaldehyde. The fixed cells were rendered permeable with 0.5% Triton X-100 in PBS and then incubated with 3% bovine serum albumin in PBS for 1 hour. Next, the cells were treated with anti-JMJD2A antibody (Abcam ab24545) at a 1 : 500 dilution overnight at 4°C, followed by three washes with PBS containing 0.01% Triton X-100, followed by the secondary antibody incubation (Alexa Fluor 488, Abcam ab150077, 1 : 500 dilution) for 1 hour. Finally, the cells were washed twice and mounted in ProLong Diamond Antifade Mountant with DAPI (Life Technologies, Eugene, OR, USA). All images were obtained on an Eclipse Ni-E microscope (Nikon) and analyzed with ImageJ software (NIH).

### 2.4. qRT-PCR

Cells were detached with lysis solution to isolate RNA using the High Pure RNA Isolation Kit (Roche Diagnostic, Indianapolis, IN, USA). The purity of the isolation was verified using agarose gels at 1.5% containing SYBR® Safe (Invitrogen, Life Technologies, Grand Island, NY, USA) and the concentration of each sample was measured using a NanoDrop (Thermo Scientific, MA, USA). cDNA was obtained using the Transcriptor First Strand cDNA Synthesis Kit (Roche Diagnostic, Indianapolis, IN, USA) and oligo(dT) primers. FastStart Essential DNA Green Master (Roche Diagnostic, Indianapolis, IN, USA) was used to examine the relative levels of mRNA through qRT-PCR. The data were normalized using GAPDH and analyzed by the ΔΔCt method. The primers used in this study were

**Table:** 

*bnp*	*F*-TCAGCCTCGGACTTGGAAAC	*R*-CTTCCAGACACCTGTGGGAC
*anp*	*F*-GACAGACTGCAAGAGGCTCC	*R*-GCTGCAGCTTAGATGGGATGA
*β-mhc*	*F*-CTGTCCAAGTTCCGCAAGGT	*R*-ATTCAAGCCCTTCGTGCCAA
*jmjd2a*	*F*-GCCGCTAGAAGTTTCAGTGAG	*R*-GCGTCCCTTGGACTTCTTATT
*jmjd2c*	*F*-GCGGTCCCAGAAGTTCGATT	*R*-TCTAGATTCCCAGCCTTCCCA
*utx*	*F*-CTCCATGGCTAGGACTGCAA	*R*-AGACACCTAACAGCACTGCC
*gapdh*	*F*-ACCCACTTCTCCACCTTTGAC	*R*-TGTTGCTGTAGCCAAATTCG

### 2.5. Western Blot Analysis

The cells were lysed after hypertrophy induction using buffer RIPA (Abcam, Cambridge, MA, USA) by adding a protein inhibitor cocktail (Roche Diagnostic, Indianapolis, IN, USA) to recover the extract. For the separation of proteins, the samples were denatured at 97.5°C for over 2 minutes and an SDS-PAGE at 12.5% was performed, after which the samples were transferred to a membrane of polyvinylidene fluoride (PVDF). The membrane was incubated in blocking buffer (TBS, 0.1% Tween-20 and 5% skimmed milk) for over 1 hour at room temperature with agitation. After, the membrane was incubated overnight at 4°C with agitation in TBS-Tween-20 solution at 0.1% v/v with primary antibodies against JMJD2A (1 : 2000) (Abcam, Cambridge, MA, USA, ab24545), BNP (1 : 500) (Abcam, Cambridge, MA, USA, ab19645), and ANP (1 : 500) (Abcam, Cambridge, MA, USA, ab76743). The membrane was then incubated for 1 hour at room temperature in TBS-Tween-20 solution at 0.1% v/v with the secondary antibody (1 : 3000) (Abcam, Cambridge, MA, USA, ab6721). Lastly, the membrane was treated with Luminata-HPR Substrate (EMD Millipore) for 5 minutes at room temperature and was visualized by chemiluminescence using MyECL (Thermo Scientific, MA, USA).

### 2.6. Transient Transfections

Functional assays to evaluate JMJD2A activity were performed using the H9C2 cell line. The cells were transfected at 80% confluence.

Plasmid DNA transfection was using Lipofectamine 2000 (Life Technologies, Carlsbad, CA, USA) with 0.5 *μ*g of pcDNA3.1-JMJD2A. The transfection mixture was prepared in 2 mL of Opti-MEM® medium (Life Technologies) with 0.5 *μ*g DNA and 0.5 *μ*L of Plus Reagent per well, with a 1 : 4 DNA : Lipofectamine ratio. After 20 minutes, the mixture was added to the cells. The medium was removed after 6 hours, replaced with complete growth medium and treated with 200 mM Ang II and 100 mM ET-1 or DMSO as a vehicle. After 24 hours, the cells were lysed and a western blot was performed to evaluate the expression levels of BNP and ANP. The results were calculated according to changes in the fetal gene products before and after the transfection compared with the empty expression vector, which was used as a control.

The gene silencing was using Lipofectamine RNAiMAX (Life Technologies, Carlsbad, CA, USA) with 100 *μ*M of siRNA. Three short/small interfering RNAs sets were tested (HSS114501, HSS190521, and HSS190522 Invitrogen, Life Technologies, Grand Island, NY, USA). The sequences of JMJD2A siRNA (sense strand and antisense strand) are as follows:

**Table:** 

*#1*	*CCGAGACCUUCUAUGAAGUCAACUU*	*AAGUUGACUUCAUAGAAGGUCUCGG*
*#2*	*AAGUUGACUUCAUAGAAGGUCUCGG*	*CCGAGACCUUCUAUGAAGUCAACUU*
*#3*	*GCCCUAGAGGAGGACUGCUGUUUAU*	*AUAAACAGCAGUCCUCCUCUAGGGC*

The transfection mixture was prepared in 250 *μ*L of Opti-MEM medium (Life Technologies) with 100 *μ*M of siRNA and 9 *μ*L of Lipofectamine per well. After 30 minutes, the mixture was added to the cells with Dulbecco's Modified Eagle Medium: Nutrient Mixture F-12 (DMEM/F-12-GlutaMAX) (GIBCO, Life Technologies, Grand Island, NY, USA) supplemented with 2% Fetal Bovine Serum (FBS) (GIBCO, Life Technologies, Grand Island, NY). After 24 hours, the cells were lysed and a western blot was performed to evaluate the expression levels of BNP. The results were calculated according to changes in the fetal gene products before and after the transfection compared with the Lipofectamine RNAiMAX without siRNA, which was used as a control.

### 2.7. Statistical Analysis

The analysis of variance test (ANOVA) was used and differences were considered statistically significant when the mean value with standard error was *p* < 0.05. Twenty-four-well plates were used, each containing approximately 2.5 × 10^5^ cells, and each cell was considered a subject of the study. Comparisons were made in triplicate.

## 3. Results

**Figure 1:**
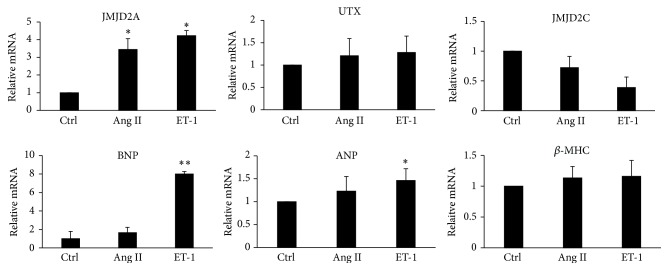
The quantitative analysis of JMJD2A, UTX, BNP, ANP, *β*-MHC, and JMJD2C expression in cardiomyocytes. Cq values of Q-PCR analysis were normalized against GAPDH. The data are presented as the mean ± SEM from at least three independent experiments. ^*∗*^ *p* < 0.05 compared with the control. ^*∗∗*^ *p* < 0.01 compared with the control.

The fetal gene expression levels that usually increase with the induction of hypertrophy were evaluated and an increase in ANP, BNP, and *β*-MHC was observed in H9C2 cells after treatment with Ang II and ET-1. In an analysis of some of the levels of histone demethylases after hypertrophic induction, an increase in JMJD2A was observed, while UTX did not change and JMJD2C levels were reduced (Figure 1).

**Figure 2:**
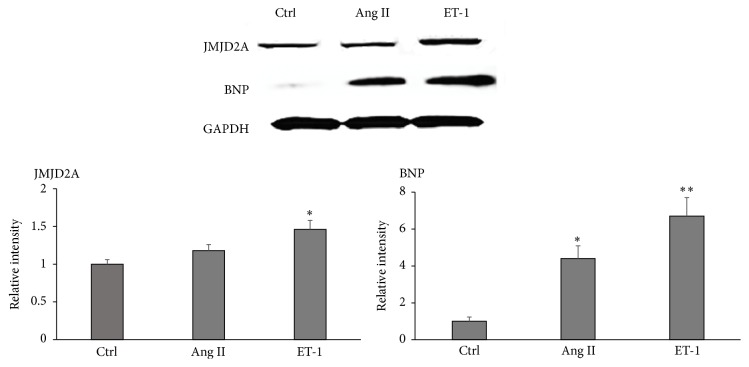
JMJD2A is upregulated in rat hypertrophic cells. The results of western blot assays using antibodies against JMJD2A and BNP with hypertrophy induced by Ang II and ET-1. GAPDH was used as the loading control. The data are presented as the mean ± SEM from at least three independent experiments. ^*∗*^ *p* < 0.05 compared with the control. ^*∗∗*^ *p* < 0.01 compared with the Ctrl (control).

Because the expression levels increased significantly in the mRNA analysis of JMJD2A after the induction of hypertrophy, JMJD2A protein levels were assessed by western blot. A significant increase in JMJD2A expression was observed when the hypertrophic induction was performed with ET-1 (Figure 2).

**Figure 3:**
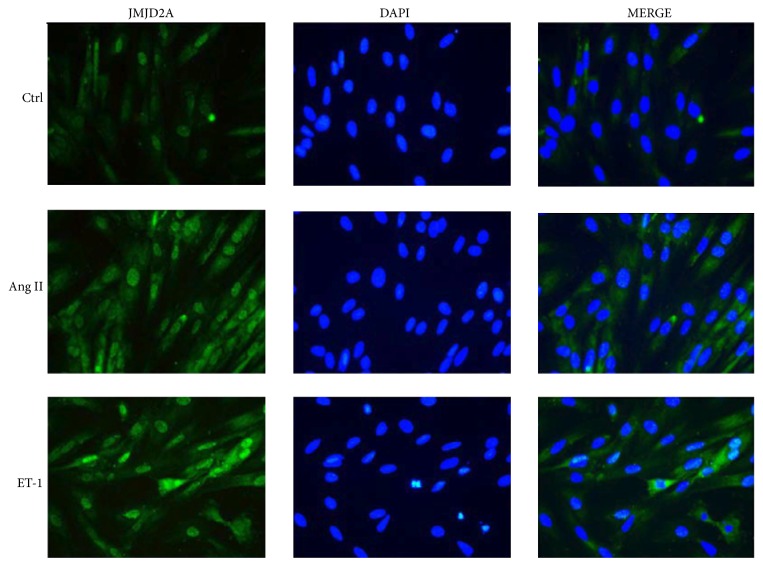
The expression and localization of JMJD2A in hypertrophic rat cells. H9C2 cells were fixed and subjected to immunofluorescence analysis using JMJD2A antibody (green). The cells were stained with DAPI (blue).

In order to recognize the impact of neurohormones on the expression and localization of JMJD2A in the cells, an immunofluorescence assessment was performed in H9C2 cardiomyocytes. It was observed that treatment with both Ang II and ET-1 increased the expression level of JMJD2A (Figure 3).

**Figure 4:**
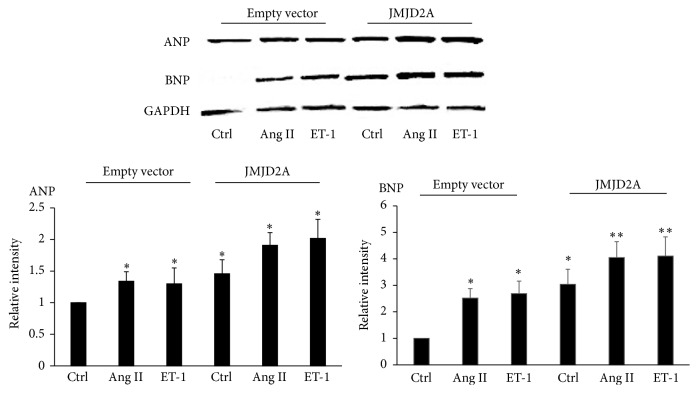
JMJD2A overexpression increased the levels of ANP and BNP. Cardiac cells were transfected at a ratio of 1 : 4 DNA : Lipofectamine, treated with Ang II 200 mM and ET-1. The results were calculated according to changes in the ANP and BNP levels after the transfection compared with the empty expression vector, which was used as a control. Data are from three independent experiments. ^*∗*^ *p* < 0.05  ^*∗∗*^ *p* < 0.01.

Since JMJD2A overexpression was evident following pharmacological treatment, we evaluated the influence of the protein on the expression levels of BNP, ANP, and *β*-MHC. After transient transfection of cardiomyocytes with a JMJD2A-containing vector, an increase in ANP and BNP was observed, which became intensified when treatment with Ang II and ET-1 was added (Figure 4).

**Figure 5:**
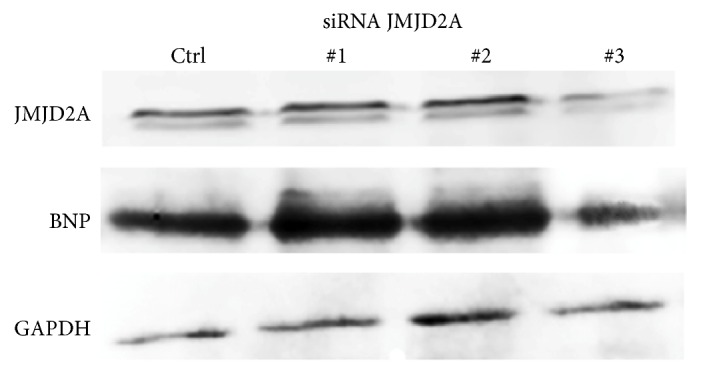
JMJD2A knockdown decreased the levels of BNP. Cardiac cells were transfected using Lipofectamine RNAiMAX and siRNA. The results of western blot assays using antibodies against JMJD2A and BNP were after 24 hours after transfection. GAPDH was used as the loading control. Data were analyzed for three different experiments which were put together.

A good way to effectively knock down gene expression to study protein function is using RNA interference. Then to further strengthen the results knockdown of JMJD2A in H9C2 cardiomyocytes was performed. After gene silencing using Lipofectamine RNAiMAX and siRNA a decrease in JMJD2A and BNP was observed. Notably, three different siRNA were used in these experiments, ruling out any off-target effects of siRNAs and the siRNA #3 showed best results in contrast with the others pairs (Figure 5).

## 4. Discussion

In this study, a variation was observed in the expression of histone demethylases in a model of hypertrophy in H9C2 rat cardiomyocytes. It is important to underscore the increased expression of JMJD2A in cardiac cells after treatment with neurohormones that cause cardiac hypertrophy. Indeed, previous studies have observed an increase in JMJD2A after cardiac hypertrophy that was induced with thoracic aortic compression (TAC) [20]. The TAC model has some limitations because the hypertrophic effect is not mediated by neurohormones, which increase in pathological cardiac hypertrophy, but has a direct mechanical effect on angiotensin receptors [14, 20, 23].

We must highlight the important role that JMJD2A performs in cardiac cells, which was shown through transfection studies by the increased levels of fetal proteins BNP and ANP in the cardiac cells in culture after the overexpression of JMJD2A. It has been shown that the level of JMJD2A is increased in patients with genetic cardiomyopathy and that the overexpression of JMJD2A in rat hearts is exacerbated after an overload of cardiac pressure induced by TAC [20]. However, it has not been possible to observe the causal process of JMJD2A protein in the development of cardiac hypertrophy.

JMJD2A and JMJD2C are histone H3K9 and H3K36 detrimethylases that belong to the same subclass. Our results showed that they have opposite expression patterns in cardiac hypertrophy. It has been demonstrated that JMJD2C is preferentially expressed in undifferentiated embryonic stem cells (ESCs) and regulates self-renewal in ESCs [24]. We previously observed the reduction of adipose cell differentiation after JMJD2C overexpression in mesenchymal progenitors; in accordance with our results it is probable that the role of JMJD2C in cardiac hypertrophic development might be limited [25]. On the other hand, UTX, like JMJD3, has a preferential H3K27 demethylase function, which has been important to the differentiation process of cardiac cells from mesodermal precursors [21, 26]. In our model, UTX was not significantly elevated during hypertrophy and it is probably because H3K27 demethylase plays a major role in the differentiation of cardiac cells from progenitor cells.

Although increased JMJD2A and BNP expression was observed with both Ang II and ET-1, it was more evident after ET-1 treatment. The ET-1 cardiac hypertrophy effect has been shown to cause stimulation of G-protein-coupled receptors resulting in the modulation of phospholipase C and the activation of cAMP pathways, which cause biochemical and structural remodeling, contributing to the development of hypertrophy [4]. ET-1 has the most predominant systemic concentration and is 10 times more powerful than Ang II, even though Ang II is important in the regulation of cardiovascular hemodynamic and structure [27].

Recent studies have related epigenetic changes in special histone demethylase modifications to several stages of cardiovascular diseases [28]. In the present study, the increase in JMJD2A observed by western blot and qRT-PCR after treatment with Ang II and ET-1 supports the theory that JMJD2A is a prohypertrophic factor in pathological conditions. An important observation is the increase of ANP and BNP after the overexpression of JMJD2A in cardiac cells, as JMJD2A may be able to regulate the expression of these fetal genes, which are responsible for the hypertrophic process. It is possible that JMJD2A participates in such adaptive responses together with other proteins present in cardiovascular diseases including HDAC4 or HP1 [29].

Moreover, the cells transfected with a siRNA directed against JMJD2A showing a decreased expression paralleled with that of BNP detected in western blot analysis. These results indicated that JMJD2A is determinant for the expression of cardiac fetal gene markers such BNP [29]. Further studies may determine the possible use of this protein as a therapeutic target in cardiac hypertrophy.

In conclusion, within the group of histone demethylases, JMJD2A plays a major role in regulating cardiac hypertrophy. Its function could be mediated by the modulation of the expression of some fetal genes involved in the pathological process of cardiac hypertrophy.
